# The Emotional Facet of Subjective and Neural Indices of Similarity

**DOI:** 10.1007/s10548-019-00743-7

**Published:** 2019-11-14

**Authors:** Martina Riberto, Gorana Pobric, Deborah Talmi

**Affiliations:** 1 Division of Neuroscience and Experimental Psychology, University of Manchester, Oxford Rd, Manchester, M13 9PL UK; 2 Department of Neurobiology, Weizmann Institute of Science, Herzl St. 234, Rehovot, Israel

**Keywords:** Emotions, Semantic memory, Similarity, Multivariate pattern analysis

## Abstract

Emotional similarity refers to the tendency to group stimuli together because they evoke the same feelings in us. The majority of research on similarity perception that has been conducted to date has focused on non-emotional stimuli. Different models have been proposed to explain how we represent semantic concepts, and judge the similarity among them. They are supported from behavioural and neural evidence, often combined by using Multivariate Pattern Analyses. By contrast, less is known about the cognitive and neural mechanisms underlying the judgement of similarity between real-life emotional experiences. This review summarizes the major findings, debates and limitations in the semantic similarity literature. They will serve as background to the emotional facet of similarity that will be the focus of this review. A multi-modal and overarching approach, which relates different levels of neuroscientific explanation (i.e., computational, algorithmic and implementation), would be the key to further unveil what makes emotional experiences similar to each other.

## Introduction

Emotional similarity refers to the similarity between the feelings that stimuli evoke in us. Poets and storytellers routinely use the power of emotional similarity to convey the emotional tone of a situation by analogy, for example, when the sadness that follows the breakup of a relationship is likened to that we feel when the weather is bad. As the famous song goes, it is ‘stormy weather, since my man and I ain’t together, keeps raining all the time…’. According to Bruner, stimuli that are very different visually and semantically may nevertheless be perceived as similar to each other because of the feelings they evoke in us (Bruner 2017). For example, we may judge an image of a homeless person begging for food and an image of a businesswoman talking on the phone as different, even if the pictures are taken at the same street corner, because one evokes a negative feeling and one a neutral feeling. On the contrary, the same image of a beggar and an image of a person injured in a car accident may be evaluated as more similar if both evoke negative feelings, even if the pictures are taken in different places around the world. In Bruner’s discussion, emotional similarity is considered an orthogonal dimension to the visual and semantic dimensions of a stimulus. Alternatively, the emotional facet of our experience of a stimulus may be considered part of its semantic meaning; in that case, emotional similarity may be reduced to a specific form of semantic similarity. This may be more appropriate when a person groups together neutral stimuli that they have experienced while the person is in the same mood. In this review, we define emotional similarity as the similarity between the emotional dimension of stimuli in the representational space. This space is in part objective and shared among individuals, and in part subjective and in continuous interaction with our experience.

The majority of research on similarity perception that has been conducted to date has focused on non-emotional stimuli, such as words, object, shapes, faces and scenes. In these studies (Goldstone et al. 1997; Golonka and Estes 2009; Greene et al. 2014; Iordan et al. 2015; King et al. 2019), participants were involved in explicit similarity judgement tasks. In others (Haxby et al. 2001; Kriegeskorte et al. 2008a, b, Haxby et al. 2011; Bruffaerts et al. 2013; Clarke and Tyler 2014; Guntupalli et al. 2016; Neyens et al. 2017), the main interest was the neural similarity, namely the similarity among neural representations associated with non-emotional stimuli during tasks not related to the similarity judgements. By contrast, less is known about what makes people perceive richer, life-like events as similar, and even less when these are emotional. Understanding the cognitive and neural mechanisms underlying emotional similarity may have implications for research on categorisation (Barrett 2004, 2017; Barsalou 2017), memory of emotional experiences (Talmi and McGarry 2012; Leal et al. 2014; Leal et al. 2018), and generalisation (Schechtman et al. 2010; Laufer and Paz 2012; Dunsmoor et al. 2013). From a clinical perspective, the study of emotional similarity could help in understanding why patients with anxiety disorders overgeneralise and judge a variety of subsequent experiences to be similar to the original fearful one (Lissek et al. 2009; Laufer et al. 2016).

Below, we review the major findings and debates in the literature on similarity, with the goal of placing the concept of ‘emotional similarity’ within the context of relevant research. With this aim, we will summarise two lines of research, one focused on explicit similarity judgements and the other on neural similarity. This is because both of them provide interesting information about what makes two stimuli similar, in terms of both cognitive dimensions and neural mechanisms. First, we will focus on semantic similarity, namely the similarity among non-emotional stimuli. We will use this literature as background for the emotional facet of the similarity, and to ask how the emotional similarity could be incorporated. Is emotional similarity a facet of semantic similarity or is a further dimension in a complex semantic space? We will end by proposing future directions in this research field.

### Semantic Similarity

We may judge two stimuli, such as a blue circle and a blue ellipse, as similar, because they share some features (the rounded shape and blue colour). Because of the number of properties that they share, we will consider them more similar than a blue ellipse and a pink square. This is line with the ‘contrast model’, which posited that similarity between two items is a function of their common features weighed against their distinctive features (Tversky 1977). The ‘contrast model is limited in that it fails to consider the relationships among features (Markman and Gentner 1993, 1994, 1997). These include thematic and taxonomic relationships, which widely contribute to semantic memory and similarity judgements (Lin and Murphy 2001; Ralph et al. 2010; Schwartz et al. 2011; Hoffman et al. 2013).

*Milk* paired with *jam* is an example of thematic relationship. Thematic relationships are defined as any temporal, spatial, causal, or functional relationships between objects, which perform complementary roles in the same scenario or event (e.g., *breakfast*) (Estes et al. 2011). It is widely known in the semantic memory literature that people judge thematically related stimuli to be more similar to each other than other stimuli (Simmons and Estes 2008; Golonka and Estes 2009; Estes et al. 2011; Chen et al. 2013). The paradigmatic stimuli are natural, complex pictures (Lang et al. 2008; Marchewka et al. 2014). For these stimuli, thematic relationships can arise from affordances (Maguire 2010), namely the possible actions that a person can perform in a specific situation. As shown by Greene et al. (2014), affordances may even be the most salient dimension in the categorisation of natural scenes. In that study, participants categorised natural complex pictures mainly according to affordance, rather than visual or taxonomic similarity (Greene et al. 2014).

*Labrador* and *Chihuahua* are taxonomically similar. While visually these animals are different (different colour, size, etc.), they are also similar, because they share some features (both bark and are four-legged), which once related bring out the category *dogs.* Thus, we group these items in the same category, *dogs*, and judge them as more similar than items from different categories (Wisniewski and Bassok 1999; Chen et al. 2013; Xiao et al. 2016; Xu et al. 2018). People also generalise these properties to new items with similar features (e.g., *German Shepherd*), and attribute to these items extra features that define the category, even if those were never directly experienced (Jackson et al. 2015). Features-based categories are organised hierarchically in semantic memory (Rosch et al. 1976). Within this hierarchy, it is often possible to distinguish between different levels: the broadest level is the superordinate (e.g., *animals*), then the basic (e.g., *dogs*) and then the subordinate (e.g., *Labrador*). Although some examples do not fit this neat classification (e.g., screwdriver or lawnmower) and there are a number of contradictory findings (Rogers and Patterson 2007; Taylor et al. 2012), many studies showed that participants are more accurate and faster in categorising objects at the basic level than at the superordinate and the subordinate level (Anglin 1977, Horton et al. 1980; Murphy and Brownell 1985; Mack et al. 2009; Iordan et al. 2015). Many of the stimuli in the emotional cognition literature have taxonomic relationships. In the IAPS set, for example, a picture of a man pointing a gun and a man wielding a knife are subordinates of the basic level ‘aggravated assault’. Emotional events are the core of our life stories and their categorisation, as well as the similarity among them, are fundamental to make them meaningful. However, most of the studies focused on the neural mechanisms underlying the perception of similarity among neutral stimuli and on the neural representations of non-emotional stimuli during cognitive and perceptual tasks.

#### Neuroimaging Studies

It is possible to map in the brain the similarity structure observed at behavioural level, by using innovative Multivariate Pattern Analysis (MVPA) methods. Among them, Representational Similarity Analysis (RSA) gained popularity in neuroscience in the last decade to investigate the cognitive and neural mechanisms of perceived similarity. This technique allows combining neural evidence with behavioural and computational data by calculating their correlation. In this way, it is possible to test whether and where the similarity structure observed at behavioural level is represented in the brain. In addition, this correlational-based technique examines the correlation between the neural representations of stimuli, as it is measured through the BOLD signal during cognitive tasks in fMRI, to draw conclusions about their similarity (Kriegeskorte et al. 2008a, b, 2012; Nili et al. 2014). In a recent MVPA study, Iordan et al. (2015) explored how the different levels of semantic categories are represented across the occipitotemporal cortex. They hypothesised that categorisation may be an emergent property of the human ventral visual system. In order to test this hypothesis, they calculated the category boundary effect as the difference between cohesiveness (within-category neural similarity) and distinctiveness (between-categories neural similarity). This quantity provides a measure of how well categories are separated at each taxonomic level. For example, at the basic level, cohesion for ‘dogs’ is defined as the average correlation between voxel activations associated with the presentation of a ‘dog’ and any other type of ‘dog’. On the other side, at the basic level, distinctiveness for ‘dogs’ is defined as average correlation between voxel activations associated with the presentation of a ‘dog’ and, for example, a ‘flower’. They found high cohesiveness in V1, such that patterns elicited by subordinates are not distinguishable. As we move up in the ventral visual stream (i.e., lateral occipital cortex, posterior middle temporal gyrus, inferior temporal gyrus), the categories become more sharply distinguishable at basic level (Iordan et al. 2015). This is in line with other studies, which showed that inferior temporal regions are involved in semantic categorisation and perceived similarity of objects (Malach et al. 1995; Martin et al. 1996; Epstein and Kanwisher 1998; Grill-Spector et al. 1998; Kriegeskorte et al. 2008a, b; Charest et al. 2014) and faces (Haxby et al. 2001, 2011; Guntupalli et al. 2016). Thus, according to these studies, semantic knowledge is not ‘located in’ one brain area, but it arises from distinct patterns of response that are distributed across brain regions (Haxby et al. 2001).

A similar perspective is reflected in the ‘hub and spoke’ model, an influential model of semantic memory. According to this model, semantic categorisation is the result of an interaction between different modality-specific cortices (i.e., the ‘spokes’) distributed across the brain, and a transmodal ‘hub’, located in the ventral part of the anterior temporal lobe (vATL) (Rogers et al. 2004; Patterson et al. 2007; Ralph et al. 2010; Lambon Ralph 2014). In particular, the ‘hub’ integrates sensory, motor and verbal information that together define a concept, and which are encoded in the different ‘spokes’. It also extracts inter-stimulus relationships that go beyond visual similarities, such as taxonomic and thematic relationships, and generalise these relationships to new stimuli with similar features. Many neuropsychological and neuroimaging findings, both in patients with semantic dementia (Bozeat et al. 2000; Nestor et al. 2006; Ralph et al. 2007; Jefferies et al. 2009; Guo et al. 2013) and in healthy controls (Pobric et al. 2007; Visser et al. 2012) support this model. The vATL interacts also with other brain regions, which are part of the semantic control (SC) network, to generate context-dependent semantic representations. This network include the posterior middle temporal gyrus, the prefrontal cortex, the intraparietal sulcus, the pre-supplementary motor area and the anterior cingulate cortex (for a review on this topic, see (Ralph et al. 2017)). Finally, as reviewed by Rice et al. (2018), the ATL is also involved in processing socially relevant semantic concepts, including person face knowledge and emotional concepts (Zahn et al. 2007, 2009; Olson et al. 2013; Collins and Olson 2014; Wang et al. 2017), because of its connection with the amygdala and orbitofrontal regions through the uncinated fasciculus (Highley et al. 2002; Von Der Heide et al. 2013). These regions might be thought as ‘emotional spokes’, which interact with the ATL to generate emotional concepts. Future studies are needed to test this hypothesis.

To summarise, semantic similarity supports core cognitive functions, such as semantic categorisation and semantic memory. Recent neuroimaging findings showed that conceptual knowledge is a widely distributed neural network, which include occipitotemporal and prefrontal regions. Different model have been proposed to explain the cognitive and neural mechanisms of semantic knowledge and similarity judgments (Riddoch et al. 1988; Damasio 1989; Caramazza et al. 1990). However, to our knowledge, these perspectives are limited to non-emotional stimuli, and have never been tested in the context of emotional similarity and categorisation.

### Emotional Similarity

While the majority of the studies about similarity judgements focused on non-emotional stimuli, a vast literature in emotion research asks what makes two emotional stimuli similar. To answer this question, participants are often asked to sort simple stimuli, such as words or faces, according to their similarity, or to rate the similarity among them on a Likert scale (Osgood 1952; Schlosberg 1952; Russell and Pratt 1980; Russell and Bullock 1985; Roberts and Wedell 1994; Halberstadt et al. 1995, 1997; Calvo and Nummenmaa 2008; Said et al. 2010; Koch et al. 2016; van Tilburg and Igou 2017). The paradigmatic finding is that participants judge the similarity according to two dimensions, the valence and the arousal of the stimuli. These dimensions are not explicitly used during the similarity judgements, but rather they represent implicit components of the cognitive structure underlying these stimuli (Barrett 2004). We can map this cognitive structure by using Multidimensional Scaling (MDS) procedure. When represented in a geometric space, defined by valence and arousal as orthogonal axes, emotional stimuli are placed along the perimeter of a circle. This is the core idea of Russell’s ‘circumplex model of emotion’ (Russell and Pratt 1980) and other dimensional theories of emotion (Mehrabian 1980; Watson and Tellegen 1985; Bradley et al. 1992; Plutchik 2001), which have been widely used in emotion research (Zevon and Tellegen 1982; Barrett and Russell 1999; Damasio 2003; Lang et al. 2008; Kuppens et al. 2013; Marchewka et al. 2014; Mäntylä et al. 2016; Yu et al. 2016). In this representational space, the distance among stimuli reflects their similarity, with short distances representing high similarity. The multi-arrangement method, a direct way to measure similarity, is based on this principle (Kriegeskorte and Mur 2012). This quick and efficient task is used for experiments with a relatively large set of stimuli, because participants simultaneously judge the similarity among many stimuli displayed together (Chikazoe et al. 2014; Chavez and Heatherton 2015), as opposed to a pairwise presentation.

Emotional similarity can be also quantified indirectly. Asking participants to rate the semantic relatedness between words (Talmi and Moscovitch 2004) or pictures (Sison and Mather 2007; Talmi et al. 2007; Gallo et al. 2009; Talmi and McGarry 2012) is an example of an indirect measure of similarity. This is because the higher the relatedness between concepts in semantic memory, the higher the similarity between them. These studies suggest that emotions increase the semantic relatedness, resulting in higher ratings among negative emotional stimuli compared to neutral ones. This might lead to a better organisation of emotional stimuli, and might explain the advantage they have in immediate memory tests (Talmi and McGarry 2012, 2013).

The findings above indicate that emotion increases perceived similarity between stimuli. Greater perceived similarity among emotional stimuli might be related to the effect of arousal on hippocampal pattern separation, the ability to store similar experiences in distinct and non-overlapping representations. This might explain why participants find it harder to discriminate between targets and similar lures when those are emotional (Segal et al. 2012; Leal et al. 2014, 2018; Mattar and Talmi 2019; Zheng et al. 2019). Other studies suggested that the arousal might also increase the generalisation among neutral stimuli during fear condition paradigms, both in healthy controls (Schechtman et al. 2010; Laufer and Paz 2012; Dunsmoor et al. 2013) and in patients with anxiety disorders (Lissek et al. 2009; Laufer et al. 2016). The generalisation is another example of indirect measure of similarity, because the higher the similarity between stimuli, the wider the generalisation between these stimuli.

#### Neuroimaging Studies

The number of neuroimaging studies in emotional similarity research is limited. To our knowledge, no neuroimaging studies have investigated neural differences in explicit judgments of similarity among the prevalent stimuli in research of emotional cognition, namely, natural, complex neutral and emotional picture scenes. Only a handful of studies have combined behavioural measures of similarity with neural data by using RSA. The results of these studies might help in understand the brain regions, which code the similarity among emotional stimuli. In these studies, during the fMRI scan participants were asked to attend to pictures while performing non-emotional rating tasks (e.g., ratings of indoor versus outdoor scenes). This was combined with behavioural judgements of similarity among the experimental stimuli. They found that brain activity patterns in regions involved in emotional processing, such as the insula and the ventromedial prefrontal cortex (VMPFC), represent the similarity structure between emotional and neutral stimuli observed at behavioural level (Chavez and Heatherton 2015; Levine et al. 2018).

Additional, indirect evidence about what make two emotional stimuli similar to each other at neural level is gleaned from neuroimaging investigations of emotional processing and categorisation. These mainly aimed at investigating how the brain codes the relationship between specific emotions, supporting either a categorical (Ekman and Friesen 1976), a dimensional (Russell and Pratt 1980), or a constructionist view (Barrett 2017). In these studies, participants were asked either to passively look at images, to attend to the feelings they evoke, to rate the valence and the arousal of these feelings, or to rate the valence and arousal of the picture and categorise it according to emotional labels (Costa et al. 2014; Ohira et al. 2006; Machajdik et al. 2010; Baucom et al. 2012; Sakaki et al. 2012; Yuen et al. 2012; Edmiston et al. 2013; den Stock et al. 2014; Motzkin et al. 2015; Hrybouski et al. 2016). The results of these studies were discrepant, probably because of the different perspectives of emotions adopted and methods used to elicit the emotions (Wager et al. 2015). In particular, locationist studies attempted to discover the unique brain feature associated with each emotional category, by adopting a one (brain region)-to-one (emotion) approach. For example, fear has been consistently localised in the amygdala (LaBar et al. 1998; LeDoux 2007; Öhman 2009), disgust in the anterior insula (Calder 2003; Wicker et al. 2003; Jabbi et al. 2008), sadness in the anterior cingulate cortex (Phan et al. 2002; Murphy et al. 2003), anger in the orbitofrontal cortex (Murphy et al. 2003; Vytal and Hamann 2010), and happiness in the dorsomedial prefrontal cortex (DMPFC) (Lindquist et al. 2012). As highlighted by Lindquist et al. (2012), supports for a locationist account would be found if instances of an emotion category (e.g., fear) are consistently and specifically associated with increased activity in a brain region (or in a set of regions within a network) across multiple published studies. However, first, many studies showed that the aforementioned regions are associated with multiple categories of emotions (Lindquist et al. 2012), and during many other sensory, perceptual and cognitive functions (Yarkoni et al. 2011; LeDoux 2012). Second, it is not clear whether the findings from the locationist literature are reliable enough or consistent across studies (Wager et al. 2015). For these reasons, a psychological constructionist approach to emotion is preferable. According to this perspective, emotions are ‘situated conceptualisations’, that is, subjective interpretations of what is happening around us. Emotions arise from the interaction among many brain regions, interconnected in large-scale networks, according to a many-to-one approach. These brain regions are implicated not only in emotional processing, but also in more ‘cognitive’ functions, such as conceptualization (simulation of previous experiences), language (representation and retrieval of semantic concepts), and executive attention (attention and working memory).

However, this represents only indirect evidence of the neurobiological underpinnings of emotional similarity. The neural mechanism that allows emotion to influence overall perceptions of similarity is still unknown, as are putative neural differences during explicit judgements of similarity between natural, complex neutral and emotional events.

#### Limitations in Emotional Similarity Literature

Although the emotional similarity literature provided interesting and relevant results, it is also limited in several important ways. First, most studies used decontextualized, simple stimuli, such as emotional faces, or words, a choice that yields more experimental control at the cost of ecological validity. This is particularly important because the known influence of context on emotional categorisation (Barrett 2017). For example, Avierez et al. (2008) observed this effect in a study about emotional categorisation, where participants were asked to indicate the category that best describes the facial expressions. They were less accurate in categorising sad faces embedded in a fearful than in a sad context: they were more likely to categorise sad faces as fearful when the faces appeared in a fearful context than when they appeared in a sad context. The same effect was observed in the categorisation of disgusted faces embedded in a pride context (Aviezer et al. 2008). Future studies in emotional similarity should adopt complex stimuli, which depict both emotional and neutral real-world scenes, such as those provided in well-validated datasets, the International Affective Picture System (IAPS) (Lang et al. 2008) and the Nencki Affective Picture System (NAPS) (Marchewka et al. 2014). So far, these more complex stimuli have seldom been used to study emotional similarity (Gallo et al. 2009; Talmi and McGarry 2012; Chikazoe et al. 2014; Chavez and Heatherton 2015; Levine et al. 2018).

As hinted above, one of the reasons that research on semantic memory and emotional similarity shied away from these more life-like picture scenes might be because there are many factors to control for during the stimuli selection. To mention some of them: the low-level visual measures (e.g., luminance, contrast, and color), the visual complexity of the pictures, the different degrees of similarity among taxonomic levels, the action(s) that the situation can afford, and the thematic similarity within emotional stimuli. In particular, as explained by Talmi and McGarry (2012), emotional stimuli are more thematically inter-related than the neutral stimuli found in validated datasets. For example, the term *car accident* may be related to *hospital*, and then to *death* in a common scenario, while neutral stimuli, such as *architecture*, *telephone* and *laundry*, are less inter-related thematically. In addition, the range of themes within the set of negative and arousing pictures (e.g. death, violence, car accidents, hospital scenes, and assaults) is reduced compared to those within the neutral images. This is also in line with higher ratings of content overlap among arousing (both positive and negative) than neutral IAPS stimuli, observed by Gallo et al. (2009).

To our knowledge, there are no studies, which controlled for all these factors, and this represent a further limitation in emotional similarity literature. For example, few recent studies have controlled complex pictures (positive, negative, neutral) for visual properties, as well as for some elements of semantic similarity—animacy (Chikazoe et al. 2014) and social/non-social (Chavez and Heatherton 2015). However, like other studies (Yuen et al. 2012; Levine et al. 2018), they did not control the stimuli for thematic similarity. For example, in the study by Chavez et al. (2015), the negative categories (i.e., social: ‘depiction of pain’ and ‘people crying’; non-social: ‘polluted water’ and ‘dirty toilet’) look more thematically related compared to neutral (i.e., social: ‘person at a computer’ and ‘person on the phone’; non-social: ‘a stack of book’ and ‘a spoon’) (Chavez and Heatherton 2015). It is relevant to control for these factors to be able to decouple the effect of emotions and of other factors (e.g., thematic similarity) on the overall perception of similarity, both at behavioural and at neural level. For example, in an unpublished pilot study, we hypothesised higher similarity ratings within 10 negative versus 10 neutral complex pictures, randomly selected from the NAPS database. The results supported our hypothesis. However, we could not conclude whether this effect was related to the emotional nature of the pictures or to a bias in the stimulus selection. This is because we did not control for the higher thematic similarity within the emotional pictures: the range of emotional themes was reduced compared to that in the neutral set. The same reasoning would be valid at neural level, if we observe higher similarity within the activity patterns in occipitotemporal regions associated with emotional than neutral stimuli. Indeed, without a method to select natural scenes in a way that is representative of their frequency in the environment it is difficult to conclude that emotional stimuli are represented as more similar at neural level than neutral stimuli. To our knowledge, no studies investigated behavioural or neural differences between neutral and emotional complex stimuli during direct similarity judgements.

#### Conclusion and Future Directions

Emotional similarity is a core construct in neuroscience, because it supports many cognitive functions, including categorization, memory, and learning. It is also involved in mechanisms underlying psychiatric conditions, such as anxiety disorders. However, very little is known about what makes us perceive real-life emotional experiences as similar. At behavioral, or computational, level, most of the studies showed that we implicitly consider the valence and the arousal as relevant dimensions during similarity judgements. Although these studies were very successful in relating behavioral and neural data using innovative MVPA, they mainly used very simple and ‘non-naturalistic’ emotional stimuli.

At neural, or implementation level, we gleaned indirect evidence about brain regions involved in emotional similarity from research on the structure of the emotional representation of complex stimuli. However, they do not explain which mechanisms lead to the activity associated with those stimuli. As suggested by Barsalou (2017), this is a common mistake in neuroscience: most of the studies related the computational and the implementation levels, ignoring the algorithmic level, namely the latent mechanisms within the ‘system’ brain ‘that performs the task’ (Barsalou 2017). Future studies should relate all these levels of explanation in MVPA emotional similarity studies, which will benefit of new and well-controlled set of stimuli. This may help in unveiling the influence of emotional similarity on the overall perception of similarity. Finally, we might discover any neural and behavioral differences in this perception between emotional and neutral real-life events, to understand whether emotional similarity is a facet of semantic similarity or a further dimension in a complex semantic space.
